# Supplementary material for: Nonhomologous tails direct heteroduplex rejection and mismatch correction during single-strand annealing in Saccharomyces cerevisiae
Source: PLoS Genet. 2024 Feb 5;20(2):e1010527. doi: 10.1371/journal.pgen.1010527 (PMC10868807; doi:10.1371/journal.pgen.1010527)
Supplement: S1 Table — (DOCX) [file pgen.1010527.s001.docx]

Supplementary Table S1. Genomic sequences of the repeated fragments.

| Name | sequence |
| --- | --- |
| A Repeat  (Right Fragment) | AGCTTTTCAATTCATC**A**TTTTTTTTTT_**A**TTCTTTTTTTTGATT**T**CGGTTTC**C**TTGAAATTTTTTTGATTCGGTAATCTCCGA**A**CAGAAGGAAGAACGAAGGAAGGAGCACAGACTTAGATTGGTATATATACGCATATGT**A**GTGTTGAAGAAACATGAAATTGCCCAGTATTCTTAACCCAACTGCACAGAACAAAAACC |
| F Repeat  (Left Fragment) | AGCTTTTCAATTCATC**T**TTTTTTTTTT**TG**TTCTTTTTTTTGATT**C**CGGTTTC**T**TTGAAATTTTTTTGATTCGGTAATCTCCGA**G**CAGAAGGAAGAACGAAGGAAGGAGCACAGACTTAGATTGGTATATATACGCATATGT**G**GTGTTGAAGAAACATGAAATTGCCCAGTATTCTTAACCCAACTGCACAGAACAAAAACC |
| FA Tailless fragment | TTTTCAATTCATC**T**TTTTTTTTTT**TG**TTCTTTTTTTTGATT**C**CGGTTTC**T**TTGAAATTTTTTTGATTCGGTAATCTCCGA**G**CAGAAGGAAGAACGAAGGAAGGAGCACAGACTTAGATTGGTATATATACGCATATGT**G**GTGTTGAAGAAACATGAAATTGCCCAGTATTCTTAACCCAACTGCACAGAACAAAAA**CCT**GCA**▽**TTTTCAATTCATC**A**TTTTTTTTTT_**A**TTCTTTTTTTTGATT**T**CGGTTTC**C**TTGAAATTTTTTTGATTCGGTAATCTCCGA**A**CAGAAGGAAGAACGAAGGAAGGAGCACAGACTTAGATTGGTATATATACGCATATGT**A**GTGTTGAAGAAACATGAAATTGCCCAGTATTCTTAACCCAACTGCACAGAACAAAAACCTGCA |
| nF Repeat  (MM2 is a T>G) | AGCTTTTCAATTCATC**T**TTTTT**G**TTT**TG**TTCTTTTTTTTGATT**C**CGGTTTC**T**TTGAAATTTTTTTGATTCGGTAATCTCCGA**G**CAGAAGGAAGAACGAAGGAAGGAGCACAGACTTAGATTGGTATATATACGCATATGT**G**GTGTTGAAGAAACATGAAATTGCCCAGTATTCTTAACCCAACTGCACAGAACAAAAACC |

Supplementary Table S1. Genomic sequences of the repeated fragments. In Tailed strains the repeated fragments are represented by two 200bp sequences. The strains with identical repeats, contain two copies of A fragment (AA strains). The strains with divergent repeats are designed as FA and present 3% homeology shown here by bolded underlined letters – red for A fragment and blue for F fragment. MM2 is represented by a T insertion on F fragment, shown here by a red underscore ( _ ) on A fragment. In Tailless strains the 200bp sequences are adjacent. The F fragment is represented by blue and the A fragment by red. The PAM sequence, CCT is shown by brown capital letters and the Cas9 cut site by a green triangle. The DSB created by Cas9 between the F and A repeated fragments, does not lead to nonhomologous tails.
