# Supplementary material for: Nonhomologous tails direct heteroduplex rejection and mismatch correction during single-strand annealing in Saccharomyces cerevisiae
Source: PLoS Genet. 2024 Feb 5;20(2):e1010527. doi: 10.1371/journal.pgen.1010527 (PMC10868807; doi:10.1371/journal.pgen.1010527)
Supplement: S2 Table — (DOCX) [file pgen.1010527.s002.docx]

Supplementary Table S2. Comparison of inducible and constitutive DSBs

Supplementary Table S2. Comparation between inducible and constitutive expression of HO and Cas9 endonucleases in strains with divergent repeats. Viabilities were investigated following unique DSBs created in an inducible way (by HO endonuclease in Tailed strains and inducible Cas9 pRT02 in Tailless strains) or in a constitutive way (by transforming the strains with Cas9 plasmids pAB101 in tailed strains and pRT01 in tailless strains) – see Materials & Methods section. Each value represents the average from at least three independent experiments. One SD of the mean is shown in parentheses. Experimental means were statistically compared by One-Way ANOVA test. ND = not determined
