# Supplementary material for: Nonhomologous tails direct heteroduplex rejection and mismatch correction during single-strand annealing in Saccharomyces cerevisiae
Source: PLoS Genet. 2024 Feb 5;20(2):e1010527. doi: 10.1371/journal.pgen.1010527 (PMC10868807; doi:10.1371/journal.pgen.1010527)
Supplement: S3 Table — (DOCX) [file pgen.1010527.s003.docx]

Supplemental Table S3. PCR assay of colonies to assess completion of SSA

|  |  |  | SSA | Parental | 2 PCR products | other |  |
| --- | --- | --- | --- | --- | --- | --- | --- |
|  | Tailless | WT FA | 100 | 0 | 0 | 0 |  |
|  |  | WT AA | 98 | 1 | 1 | 0 |  |
|  |  | *rad52* FA | 95 | 4 | 1 | 0 |  |
|  |  | rad52 AA | 100 | 0 | 0 | 0 |  |
|  | Two-tail, *GAL*::*HO* | WT FA | 100 | 0 | 0 | 0 |  |
|  |  | WT AA | 100 | 0 | 0 | 0 |  |
|  |  | *rad52* AA | 14 | 71 | 0 | 14 |  |
|  |  | *rad52* FA | 54 | 42 | 0 | 4 |  |
|  | Two-tail, Tailless target Cas9 DSB-2 | WT FA | 65 | 0 | 35 | 0 |  |
|  |  | WT AA | 88 | 5 | 7 | 0 |  |
|  |  | *rad52* AA | 9 | 81 | 0 | 9 |  |
|  |  | *rad52* FA | 9 | 87 | 0 | 4 |  |
|  | Two-tail, HOcs target Cas9 DSB-1 | WT FA | 100 | 0 | 0 | 0 |  |
|  |  | WT AA | 100 | 0 | 0 | 0 |  |
|  |  | *rad52* FA | 48 | 52 | 0 | 0 |  |
|  |  | *rad52* AA | 81 | 19 | 0 | 0 |  |
|  | Two-DSBs | WT AA | 99 | 0 | 1 | 0 |  |
|  |  | *rad52* AA | 50 | 0 | 50 | 0 |  |
|  | One-tail | WT AA R-DSB | 98 | 0 | 2 | 0 |  |
|  |  | WT AA L-DSB | 98 | 0 | 2 | 0 |  |
|  |  | WT FA R-DSB | 92 | 0 | 8 | 0 |  |
|  |  | WT FA L-DSB | 96 | 0 | 4 | 0 |  |
|  |  | *rad52* AA R-DSB | 74 | 0 | 26 | 0 |  |
|  |  | *rad52* AA L-DSB | 79 | 21 | 0 | 0 |  |
|  |  | *rad52* FA R-DSB | 75 | 0 | 25 | 0 |  |
|  |  | *rad52* FA L-DSB | 62 | 12 | 24 | 2 |  |
|  | Plasmid Transformation | WT *Eco*RI | 100 | 0 | 0 | 0 |  |
|  |  | WT *Hind*III | 100 | 0 | 0 | 0 |  |
|  |  | WT *Hind*III+*Sph*I | 100 | 0 | 0 | 0 |  |
|  |  | WT *Sph*I | 100 | 0 | 0 | 0 |  |
|  |  | *rad52* *Eco*RI | 43 | 57 | 0 | 0 |  |
|  |  | *rad52* *Hin*dIII | 33 | 67 | 0 | 0 |  |
|  |  | *rad52* *Hin*dIII+*Sph*I | 100 | 0 | 0 | 0 |  |
|  |  | *rad52* *Sph*I | 77 | 23 | 0 | 0 |  |
|  |  |  |  |  |  |  |  |

Supplemental Table S3. Colony PCR results of galactose-induced colonies when analyzed by PCR (see Materials and Methods) shown as percentages.  A PCR product of 285bp was produced if an SSA product was present; 632bp if the parental configuration was retained.  Both products were sometimes observed (2 PCR products). PCR products of other sizes were not investigated further.
