# Supplementary material for: Nonhomologous tails direct heteroduplex rejection and mismatch correction during single-strand annealing in Saccharomyces cerevisiae
Source: PLoS Genet. 2024 Feb 5;20(2):e1010527. doi: 10.1371/journal.pgen.1010527 (PMC10868807; doi:10.1371/journal.pgen.1010527)
Supplement: S4 Table — (DOCX) [file pgen.1010527.s004.docx]

Supplementary Table S4. Yeast strains

| Yeast Strain | Genotype | Comments |
| --- | --- | --- |
|  | **2-tail strains** |  |
| tNS1357 | *ho HML*α *mat*Δ::*leu2*::hisG *hmr-3*Δ *mal2 leu2 trp1 thr4* (*THR4* *ura3-F*(205 bp) HOcs *URA3-A*) pFH800 (*GAL::HO TRP1 CEN*) | FA Tailed strain |
| tNS1379 | *ho HML*α *mat*Δ::*leu2*::hisG *hmr-3*Δ *mal2 leu2 trp1 thr4* (*THR4* *ura3- A*(205 bp) HOcs *URA3*) pFH800 (*GAL::HO TRP1 CEN*) | AA Tailed strain |
| tNS1357t | ho *HML*α *mat*Δ::*leu2*::hisG *hmr*-*3*Δ *mal2 leu2 trp1 thr4* (*THR4* *ura3-F* (205 bp) HOcs *ura3*-A) | FA Tailed strain |
| tNS1379t | *ho HML*α *mat*Δ::*leu2*::hisG *hmr-3*Δ *mal2 leu2 trp1 thr4* (*THR4 ura3-A* (205 bp) HOcs *URA3*) | AA Tailed strain |
| tNS2892 | tNS1357t with tailless DSB (*Gal::*Cas9 DSB-2) pRT2 (*GAL*::Cas9 *LEU2*) | FA Cas9 DSB-2 |
| tNS2893 | tNS1379t with tailless DSB (*Gal::*Cas9 DSB-2) pRT2 (*GAL*::Cas9 *LEU2*) | AA Cas9 DSB-2 |
| tNS2901 | tNS2892 *rad52*::KANMX | FA *rad52* Cas9 DSB-2 |
| tNS2902 | tNS2893 *rad52*::KANMX | AA *rad52* Cas9 DSB-2 |
| tNS2910 | tNS1357t *hml*::NATMX pNSU318 (*Gal::*Cas9 targeting Cas9 DSB-1) | FA Cas9 DSB-1 |
| tNS2911 | tNS1379t *hml*::NATMX pNSU318 (*Gal::*Cas9 targeting Cas9 DSB-1) | AA Cas9 DSB-1 |
| tNS2917 | tNS2911 *rad52*::KANMX | AA *rad52* Cas9 DSB-1 |
| tNS2918 | tNS2910 *rad52*::KANMX | FA *rad52* Cas9 DSB-1 |
| YES08  (EAY997) | tNS1379 *sgs1*::KANMX6 | AA Tailed *sgs1*::KAN |
| YES09  (EAY994) | tNS1357 *sgs1*::KANMX6 | FA Tailed *sgs1*::KAN |
| YES12 | tNS1357 *rad1*::KANMX6 | FA Tailed *rad1*::KAN |
| YES13 | tNS1379 *rad1*::KANMX6 | AA Tailed *rad1*::KAN |
| YES93 | tNS1357 *pms1*::*LEU2* | FA Tailed *pms1*::*LEU2* |
| YES94 | tNS1357 *mlh1*::*LEU2* | FA Tailed *mlh1*::*LEU2* |
| YES95 | tNS1357 *msh6*::KANMX | FA Tailed *msh6*::KAN |
| YES96 | tNS1357 *exo1*::KANMX | FA Tailed *exo1*::KAN |
| YES97 | tNS1357 *srs2*::KANMX | FA Tailed *srs2*::KAN |
| tNS1815 | tNS1357 *msh2*::KANMX | FA Tailed *msh2*::KAN |
| YES130 | tNS1379 *msh3*::*LEU2* | AA Tailed *msh3*::*LEU2* |
| YES132 | tNS1379 *msh2*::*LEU2* | AA Tailed *msh2*::*LEU2* |
| YES134 | tNS1379 *rad51*::*LEU2* | AA Tailed *rad51*::*LEU2* |
| YES135 | tNS1379 *rad52*::KANMX | AA Tailed *rad52*::KAN |
| YES136 | tNS1357 *rad52*::HPHMX | FA Tailed r*ad52*::HPH |
| YES138 | tNS1357 *msh3*::HPHMX | FA Tailed *msh3*::HPH |
| YES154 | tNS1357 *msh6*::KAN *msh3*::HPHMX | FA Tailed double mutant |
| YES157 | tNS1357 *pol3-01* | FA Tailed *pol3*-*01* |
| YES179 | tNS1357 *msh2*::KAN *msh6*::NATMX | FA Tailed double mutant |
| YES34 | tNS1357 with mutated F fragment | nFA Tailed strain |
| YES49 | *ho HML*α *mat*Δ::*leu2*::hisG *hmr-3*Δ *mal2 leu2 trp1 thr4* (*THR4* *URA3-A* (205 bp) HOcs *ura3-F* pFH800 (*GAL::HO TRP1 CEN*) | AF Tailed strain |
| YES65 | YES49 with mutated F fragment | AnF Tailed strain |
|  |  |  |
|  | **Tailless strains** |  |
| yRT03 | *ho HML*α *mat*Δ::*leu2*::hisG *hmr-3*Δ *mal2 leu2 trp1 thr4* (*THR4 ura3-F*(205 bp) *URA3-A* Tailless SSA) pFH800 (*GAL::HO TRP1 CEN*) pRT2 (*GAL*::Cas9 *LEU2*) | FA Tailless strain |
| yRT04 | *ho HML*α *mat*Δ::*leu2*::hisG *hmr-3*Δ *mal2 leu2 trp1 thr4* (*THR4 ura3-A* (205 bp) *URA3-A* Tailless SSA) pFH800 (*GAL::HO* *TRP1 CEN*) pRT2 (*GAL*::Cas9 *LEU2*) | AA Tailless strain |
| YES24 | yRT03 *sgs1*::NATMX | FA Tailless *sgs1*::NAT |
| YES118 | yRT03 *msh2*::KANMX | FA Tailless *msh2*::KAN |
| YES119 | yRT03 *pms1*::*LEU2* | FA Tailless *pms1*::LEU |
| YES120 | yRT03 *srs2*::KANMX | FA Tailless *srs2*::KAN |
| YES121 | yRT03 *exo1*::KANMX | FA Tailless *exo1*::KAN |
| YES122 | yRT03 *msh6*::KANMX | FA Tailless *msh6*::KAN |
| tNS2874 | yRT03 *msh3*::KANMX | FA Tailless *msh3*::KAN |
| tNS2875 | yRT04 *msh3*::KANMX | AA Tailless *msh3*::KAN |
| tNS2871 | yRT03 *rad52*::KANMX | FA Tailless *rad52*::KAN |
| tNS2872 | yRT04 *rad52*::KANMX | AA Tailless *rad52*::KAN |
|  |  |  |
|  | **2 DSBs strains** |  |
| YES177 | tNS1379t with 2 Cas9 DSBs adjacent to each repeat | AA 2 PAMs |
| YES186 | YES177 pES57 (*GAL*::Cas9 2-gRNAs HPHMX) | AA 2 DSBs |
| YES194 | tNS1357t with 2 *GAL*::Cas9 DSBs adjacent to each repeat | nFA 2 PAMs |
| tNS2873 | YES186 *rad52*::KANMX | AA 2 DSBs |
|  |  |  |
|  | **1-tail strains** |  |
| tNS2876 | YES177 pES55 (*GAL*::Cas9 HPHMX) | AA 1-tail right DSB |
| tNS2877 | YES177 pES56 (*GAL*::Cas9 HPHMX) | AA 1-tail left DSB |
| tNS2878 | YES194 pES55 (*GAL*::Cas9 HPHMX) | FA 1-tail right DSB |
| tNS2879 | YES194 pES56 (*GAL*::Cas9 HPHMX) | FA 1-tail left DSB |
| tNS2880 | YES177 *rad52*::KANMX pES55 (*GAL*::Cas9 HPHMX) | AA 1-tail *rad52* right DSB |
| tNS2881 | YES177 *rad52*::KANMX pES56 (*GAL*::Cas9 HPHMX) | AA 1-tail *rad52* left DSB |
| tNS2882 | YES194 *rad52*::KANMX pES55 (*GAL*::Cas9 HPHMX) | FA 1-tail *rad52* right DSB |
| tNS2883 | YES194 *rad52*::KANMX pES56 (*GAL*::Cas9 HPHMX) | FA 1-tail *rad52* left DSB |
