# Supplementary material for: Nonhomologous tails direct heteroduplex rejection and mismatch correction during single-strand annealing in Saccharomyces cerevisiae
Source: PLoS Genet. 2024 Feb 5;20(2):e1010527. doi: 10.1371/journal.pgen.1010527 (PMC10868807; doi:10.1371/journal.pgen.1010527)
Supplement: S5 Table — (DOCX) [file pgen.1010527.s005.docx]

Supplementary Table S5. Plasmids

| Name | Description | backbone |
| --- | --- | --- |
| pFH800 | *GAL*::*HO* *TRP1 CEN4 ARS1* | pUC19 |
| pJH727 | *GAL::HO* *LEU2 CEN4 ARS1* | YCp50 |
| pAB101 | Cas9 plasmid targeting *MAT*a locus (Cas9 DSB-1) | bRA89 |
| pES02 | Cas9 plasmid that cuts at middle distance between the two repeated fragments | bRA89 |
| pES6 | Cas9 plasmid that cuts upstream F repeat; used to create nFA strains | bRA89 |
| pES08 | Cas9 plasmid that cuts 24 bp upstream of the Right fragment; used to insert a PAM adjacent to Right fragment | bRA89 |
| pES11 | Cas9 plasmid that cuts downstream A fragment; used to create the AF strain from AA strain | bRA89 |
| pES18 | Cas9 plasmid that cuts downstream F repeat – used to insert a PAM adjacent to Left Fragment | bRA90 |
| pES19 | Cas9 plasmid that cuts adjacent to Left repeat | bRA89 |
| pES20 | Cas9 plasmid that cuts adjacent to Right repeat | bRA89 |
| pRT01 | Cas9 plasmid that cuts between the *ura3* repeats | bRA90 |
| pRT02 | *GAL*::Cas9 carried on a centromeric Leu plasmid | bRA77 |
| pNSU318 | *GAL*::Cas9 plasmid targeting *MAT*a locus (Cas9 DSB-1) HPHMX | bRA66 |
| pNSU319 | 200-bp repeats (AA), *CEN6* *ARS* *URA3* | pNSU118 |
| pES53 | *HPHMX* Cas9 plasmid with 2 gRNAs | bRA66 |
| pES55 | *HPHMX* Cas9 plasmid and engineered for the generation of a DSB proximal to the Right repeat | pES53 |
| pES56 | *HPHMX* Cas9 plasmid and engineered for the generation of a DSB proximal to the Left repeat | pES53 |
| pES57 | *HPHMX* Cas9 plasmid engineered for the concurrent induction of two DSBs proximal to the Left and the Right repeats | pES56 |
