# Supplementary material for: Nonhomologous tails direct heteroduplex rejection and mismatch correction during single-strand annealing in Saccharomyces cerevisiae
Source: PLoS Genet. 2024 Feb 5;20(2):e1010527. doi: 10.1371/journal.pgen.1010527 (PMC10868807; doi:10.1371/journal.pgen.1010527)
Supplement: S6 Table — (DOCX) [file pgen.1010527.s006.docx]

Supplementary Table S6. Oligos and primers

| Oligo Number | Sequence | Comments |
| --- | --- | --- |
| NS150-*ura3* | GAAATTGCCCAGTATTCTTAACCCAACTGCACAGAACAAAAACCtgcaTTTTCAATTCATCaTTTTTTTTTTaTTCTTTTTTTTGATTtCGGTTTCcTTG | Template to build Tailless strain |
| NS151-*ura3* | CAAgGAAACCGaAATCAAAAAAAAGAAtAAAAAAAAAAtGATGAATTGAAAAtgcaGGTTTTTGTTCTGTGCAGTTGGGTTAAGAATACTGGGCAATTTC | Template to build Tailless strain |
| NS398-HOcs | TCTACAAAACCAAAACCAGGGTTTATAAAATTATACTGTTAAATGATGAATTGAAAATGCAGGTGTTGCGGAAAGCTGAAACTAAAAGAAAAACCCGACTATG | Template to insert the Cas9 tailless target sequence into the HOcs sequence. |
| NS154-pUC | GGTGTGAAATACCGCACAGATG | FW primer to screen for SSA |
| URA3p14 | GTAGCTTTCGACATGATTTATCTTCGTT | REV primer to screen for SSA |
| pUCp1 | CGCACAGATGCGTAAGGAGAAA | FW primer to screen DSB survivors |
| 102-1 | AGTCAGCTGCGTCGTTTG | REV primer to screen DSB survivors |
| ES148_NGS_barcodes_FW | ACACTCTTTCCCTACACGACGCTCTTCCGATCTactggtctGGTGTGAAATACCGCACAGATG | NGS FW primer to screen for SSA |
| ES149_NGS_FW_2 | ACACTCTTTCCCTACACGACGCTCTTCCGATCTaggctcaaGGTGTGAAATACCGCACAGATG | NGS FW primer to screen for SSA |
| ES150_NGS_FW_3 | ACACTCTTTCCCTACACGACGCTCTTCCGATCTatggaacgGGTGTGAAATACCGCACAGATG | NGS FW primer to screen for SSA |
| ES151_NGS_FW_4 | ACACTCTTTCCCTACACGACGCTCTTCCGATCTcagaacgtGGTGTGAAATACCGCACAGATG | NGS FW primer to screen for SSA |
| ES152_NGS_FW_5 | ACACTCTTTCCCTACACGACGCTCTTCCGATCTcgcgactaGGTGTGAAATACCGCACAGATG | NGS FW primer to screen for SSA |
| ES153_NGS_FW_6 | ACACTCTTTCCCTACACGACGCTCTTCCGATCTcgtcttggGGTGTGAAATACCGCACAGATG | NGS FW primer to screen for SSA |
| ES154_NGS_FW_7 | ACACTCTTTCCCTACACGACGCTCTTCCGATCTtcacggatGGTGTGAAATACCGCACAGATG | NGS FW primer to screen for SSA |
| ES155_NGS_FW_8 | ACACTCTTTCCCTACACGACGCTCTTCCGATCTtggtgagaGGTGTGAAATACCGCACAGATG | NGS FW primer to screen for SSA |
|  |  |  |
| ES149_NGS_BARCODES_REV | GACTGGAGTTCAGACGTGTGCTCTTCCGATCTaacacatcGTAGCTTTCGACATGATTTATCTTCGTT | NGS REV primer to screen for SSA |
| ES156_NGS_REV_2 | GACTGGAGTTCAGACGTGTGCTCTTCCGATCTagcaggatGTAGCTTTCGACATGATTTATCTTCGTT | NGS REV primer to screen for SSA |
| ES157_NGS_REV_3 | GACTGGAGTTCAGACGTGTGCTCTTCCGATCTcggtttgaGTAGCTTTCGACATGATTTATCTTCGTT | NGS REV primer to screen for SSA |
| ES158_NGS_REV_4 | GACTGGAGTTCAGACGTGTGCTCTTCCGATCTctaatgcgGTAGCTTTCGACATGATTTATCTTCGTT | NGS REV primer to screen for SSA |
| ES159_NGS_REV_5 | GACTGGAGTTCAGACGTGTGCTCTTCCGATCTctgccttcGTAGCTTTCGACATGATTTATCTTCGTT | NGS REV primer to screen for SSA |
| ES160_NGS_REV_6 | GACTGGAGTTCAGACGTGTGCTCTTCCGATCTcttggccaGTAGCTTTCGACATGATTTATCTTCGTT | NGS REV primer to screen for SSA |
| ES161_NGS_REV_7 | GACTGGAGTTCAGACGTGTGCTCTTCCGATCTgacttagtGTAGCTTTCGACATGATTTATCTTCGTT | NGS REV primer to screen for SSA |
| ES162_NGS_REV_8 | GACTGGAGTTCAGACGTGTGCTCTTCCGATCTgagcagtaGTAGCTTTCGACATGATTTATCTTCGTT | NGS REV primer to screen for SSA |
| ES163_NGS_REV_9 | GACTGGAGTTCAGACGTGTGCTCTTCCGATCTgctggatcGTAGCTTTCGACATGATTTATCTTCGTT | NGS REV primer to screen for SSA |
| ES164_NGS_REV_10 | GACTGGAGTTCAGACGTGTGCTCTTCCGATCTggaacctaGTAGCTTTCGACATGATTTATCTTCGTT | NGS REV primer to screen for SSA |
| ES165_NGS_REV_11 | GACTGGAGTTCAGACGTGTGCTCTTCCGATCTtcccaaccGTAGCTTTCGACATGATTTATCTTCGTT | NGS REV primer to screen for SSA |
| ES166_NGS_REV_12 | GACTGGAGTTCAGACGTGTGCTCTTCCGATCTtgagagctGTAGCTTTCGACATGATTTATCTTCGTT | NGS REV primer to screen for SSA |
| ES25 | cgtaaggagaaaataccgcatcaagcccggatccgagc TTTTCAATTCATCTTTTTTTGTTTGTTCTTTTTTTTGATTCCGGTTT | 90mer mutated template to remove the T insertion from F-*URA3* in tNS1357 strain; The MM2 is represented by a G instead of a T. |
| ES28 | TTGGTAACCTGACAAAGGTTAAATTAGAAAAGCTTTTCAATTCAATTCATCATTTTTTTT | FW-Mixed oligo to amplify F fragment from tNS1357 strain by 2 steps PCR; the PCR fragment is used to build the AF strain |
| ES46-REV-F-amplif | TTATATGTAGCTTTCGACATGATTTATCTTCGTTTAATGCAGGTTTTTGTTCTGTGCAGTT | REV-Mixed oligo to amplify F fragment from tNS1357 strain by 2 steps PCR; the PCR fragment is used to build the AF strain in the presence of a CAS9 pES11 that cuts upstream right A fragment – Use with ES28 |
| ES67 | TCTTAACCCAACTGCACAGAACAAAAACCCAGAGGTCCGCGCCATTCGCCATTCAGGCTGCGCAACTGTTAAGGGCGATCGGTGCGGGC | Oligo template to insert a PAM sequence downstream Left_*URA3* fragment in order to create a DSB that leads to one nonhomologous tail |
| ES114-Right-PAM | CGACAGGACACACCCTCTTGAGCCTACAACCTTAGTAGTTGGTAACCTGACAAGTTAAATTAccGAAAAGCTTTTCAATTCA | Oligo template to insert a PAM sequence upstream Right_*URA3* fragment in order to create a DSB that leads to only one nonhomologous tail |
| NS403-Cen | CATCAGGCGCGGATCCcacgtgctataaaaataattataatttaaattttttaatataaa | Oligo used to insert CEN6-ARS into pNSU118 to create pNSU319 |
| NS402-ura3 | TATGGCGGAAAGGTATGCATGCCCTaggtttttgttctgtgcagtt | Oligo used to insert CEN6-ARS into pNSU118 to create pNSU319 |
