# Supplementary material for: Nonhomologous tails direct heteroduplex rejection and mismatch correction during single-strand annealing in Saccharomyces cerevisiae
Source: PLoS Genet. 2024 Feb 5;20(2):e1010527. doi: 10.1371/journal.pgen.1010527 (PMC10868807; doi:10.1371/journal.pgen.1010527)
Supplement: S7 Table — (DOCX) [file pgen.1010527.s007.docx]

Supplementary Table S7. Primers to assembly gRNAs

| GM207 | TTTATAAAATTATACTGTTGGTTTT | Forward primer to assemble a gRNA into Cas9 (bRA89) plasmids that targets the HO cut site; to build pAB101 plasmid |
| --- | --- | --- |
| GM208 | CAGTATAATTTTATAAACCCGATCA | Forward primer to assemble a gRNA into Cas9 (bRA89) plasmids that targets the HO cut site; to build pAB101 plasmid |
| ES4 | ACGAACATTCATGCTTTTATGTTTT | FW oligo to clone pES02 CAS9 plasmid with a gRNA directed to cut at middle distance between the two repeated fragments, in lambda DNA |
| ES5 | ATAAAAGCATGAATGTTCGTGATCA | FW oligo to clone pES02 CAS9 plasmid with a gRNA directed to cut at middle distance between the two repeated fragments, on lambda DNA |
| ES23 | AGAAAATACCGCATCAGGCGGTTTT | FW oligo to clone a CAS9 (pES06) that cuts upstream F fragment from tNS1357 strain |
| ES24 | CGCCTGATGCGGTATTTTCTGATCA | Rev oligo to clone a CAS9 (pES06) that cuts upstream F fragment from tNS1357 strain |
| ES34 | AGTAGTTGGTAACCTGACAAGTTTT | FW oligo to clone a CAS9 (pES8) that cuts 24 bp upstream Right A fragment in Tailed strains |
| ES35 | TTGTCAGGTTACCAACTACTGATCA | REV oligo to clone a CAS9 (pES8) that cuts 24 bp upstream Right A fragment in Tailed strains |
| ES44 | TGCACAGAACAAAAACCTGCGTTTT | FW oligo to clone a CAS9 (pES11) that cuts downstream A fragment form tNS1379 strain |
| ES45 | GCAGGTTTTTGTTCTGTGCAGATCA | REV oligo to clone a CAS9 (pES11) that cuts downstream A fragment from tNS1379 strain |
| ES65 | ATTCAGGCTGCGCAACTGTTGTTTT | FW oligo to clone pES18 CAS9 that specifically cuts downstream Left_*URA3* fragment; use it to insert a PAM seq that allows creation of a DSB leading to only one nonhomologous tail |
| ES66 | AACAGTTGCGCAGCCTGAATGATCA | REV oligo to clone pES18 CAS9 that specifically cuts Downstream Left_*URA3* fragment; use it to insert a PAM seq that allows creation of a DSB leading to only one nonhomologous tail |
| ES68 | CTGCACAGAACAAAAACCCAGGTTT | FW oligo to clone the pES19 CAS9 that specifically cuts adjacent F (Left) fragment; only one nonhomologous tail will result after the DSB is created by pES19 |
| ES69 | CTGGGTTTTTGTTCTGTGCAGGATCA | REV oligo to clone the pES19 CAS9 |
| ES201 | AAAAGCTTTTCAATTCATCAGATCA | FW oligo to clone the pES20 Cas9 and its inducible form pES55 that specifically cuts adjacent A (Right) fragment; only one nonhomologous tail will result after the DSB created by pES20 or by pES55 |
| ES202 | ATGATGAATTGAAAAGCTTTTGTTTT | FW oligo to clone the pES20 and pES55 Cas9 plasmids |
| ES209 | GAGTTACTGTCTGTTTTCCTCTGCACAGAACAAAAACCCAGGTTTCAGAGCTATGCTGGAA | FW Oligo to clone a gRNA into pES53 gal inducible plasmid digested with *Not*I. This gRNA targets a region near Left fragment in SSA strains, so the resulting Cas9 (pES56) cuts adjacent Letf-*URA3* fragment and NO nonhomologous tail will result after the DSB; duplex it with ES210 |
| ES210 | TTCCAGCATAGCTCTGAAACCTGGGTTTTTGTTCTGTGCAGAGGAAAACAGACAGTAACTC | Rev Oligo to generate pES56 Cas9 that creates a DSB adjacent Left-*URA3*; duplex it with ES209 |
