# Supplementary figures and images for: Nonhomologous tails direct heteroduplex rejection and mismatch correction during single-strand annealing in Saccharomyces cerevisiae
Source: PLoS Genet. 2024 Feb 5;20(2):e1010527. doi: 10.1371/journal.pgen.1010527 (PMC10868807; doi:10.1371/journal.pgen.1010527)

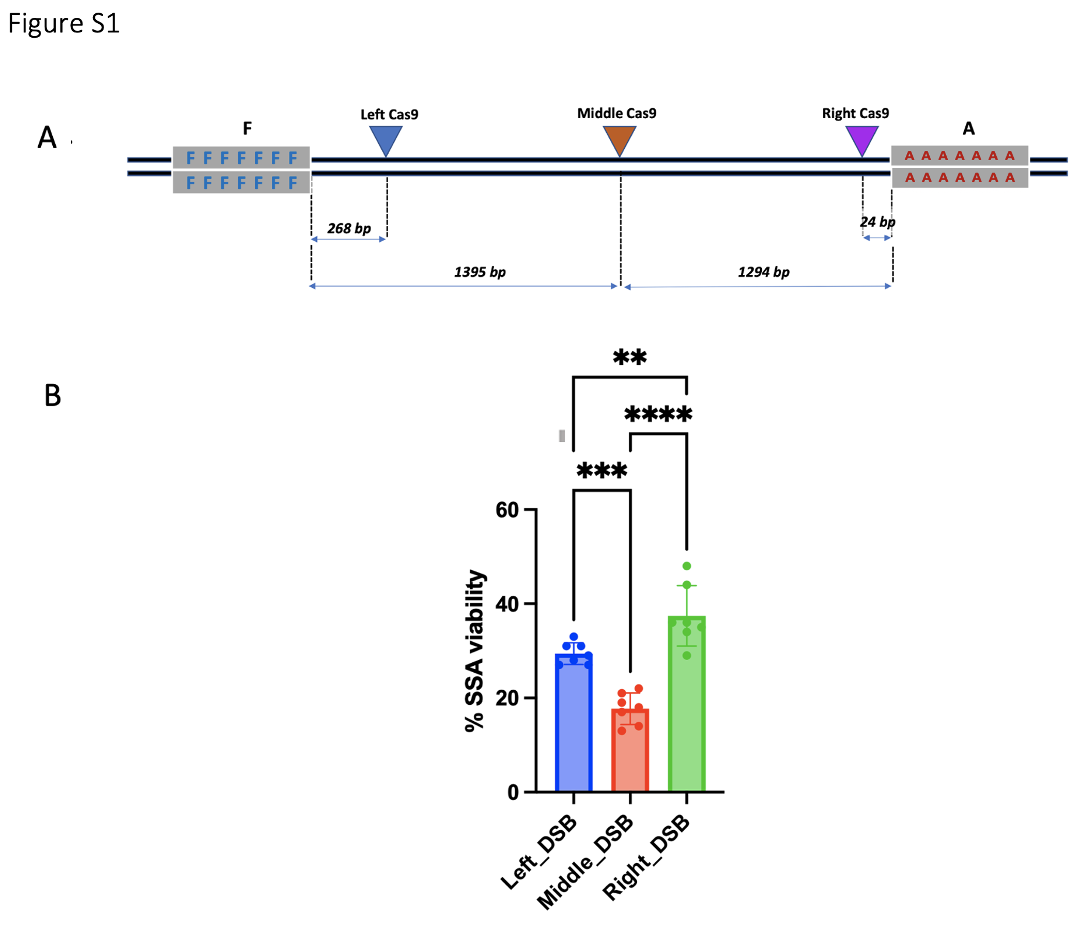

Supplement: S1 Fig — (TIF) [file pgen.1010527.s008.tif]

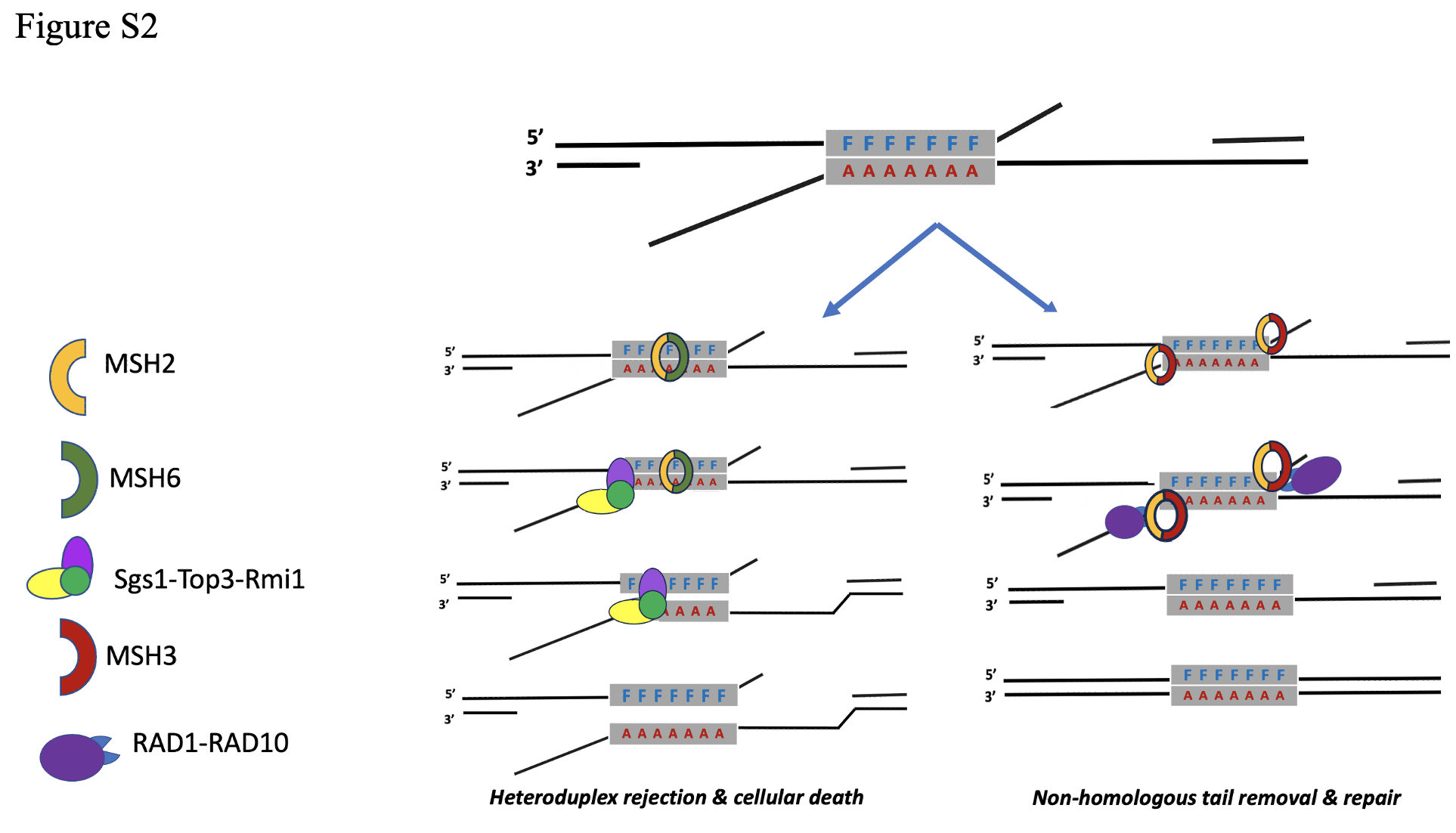

Supplement: S2 Fig — (TIF) [file pgen.1010527.s009.tif]

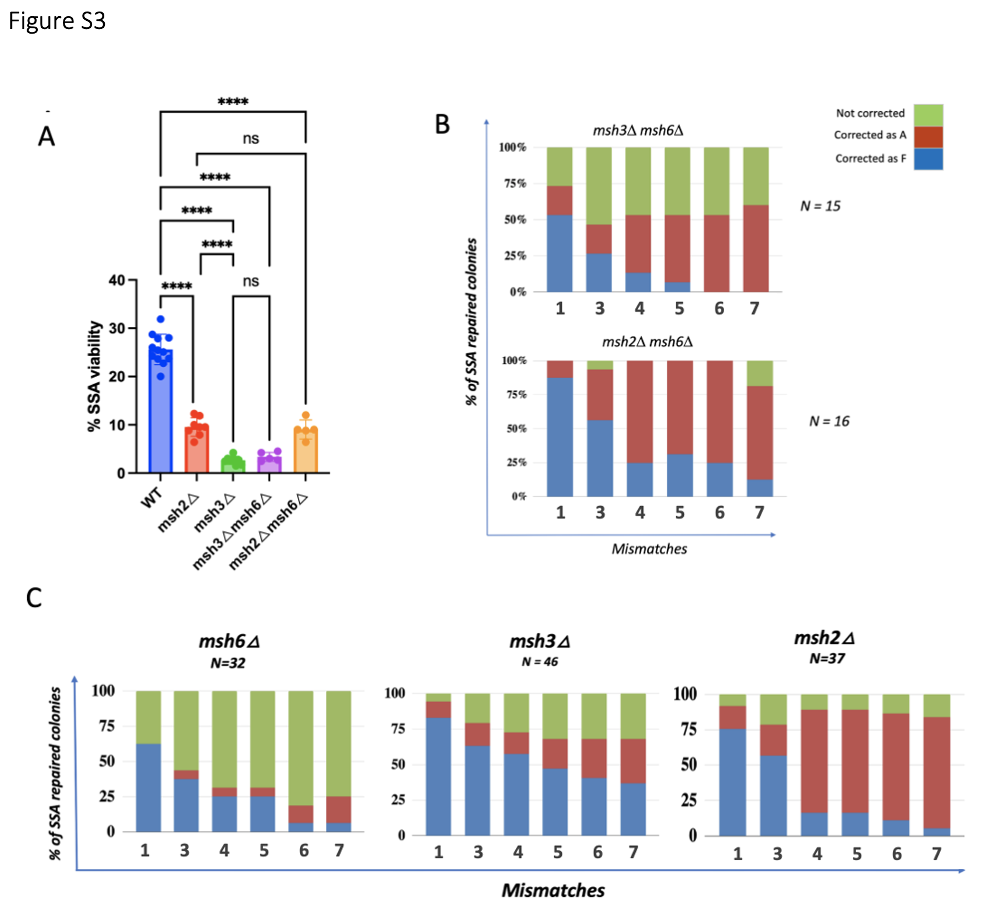

Supplement: S3 Fig — (TIF) [file pgen.1010527.s010.tif]

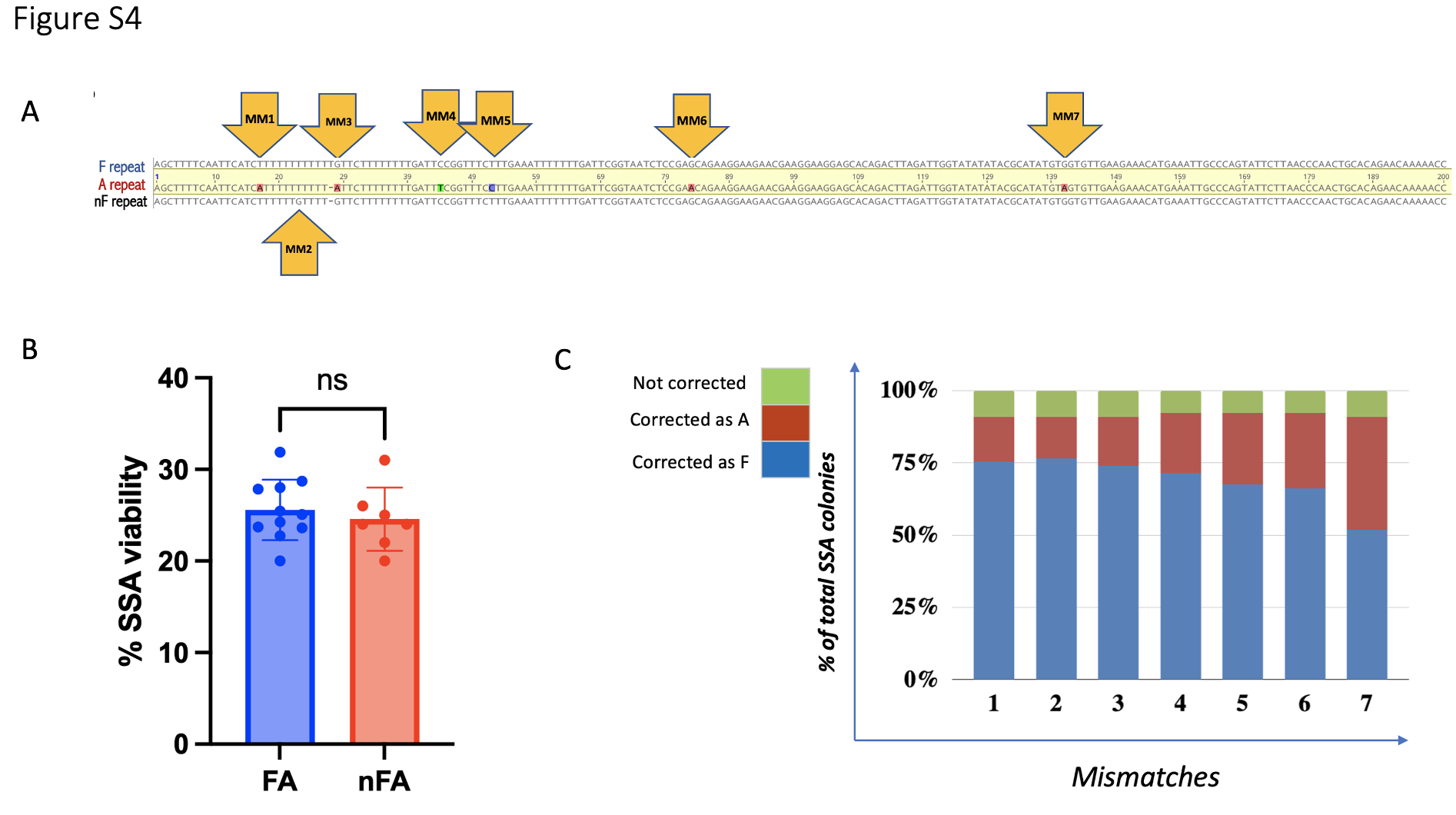

Supplement: S4 Fig — (TIF) [file pgen.1010527.s011.tif]

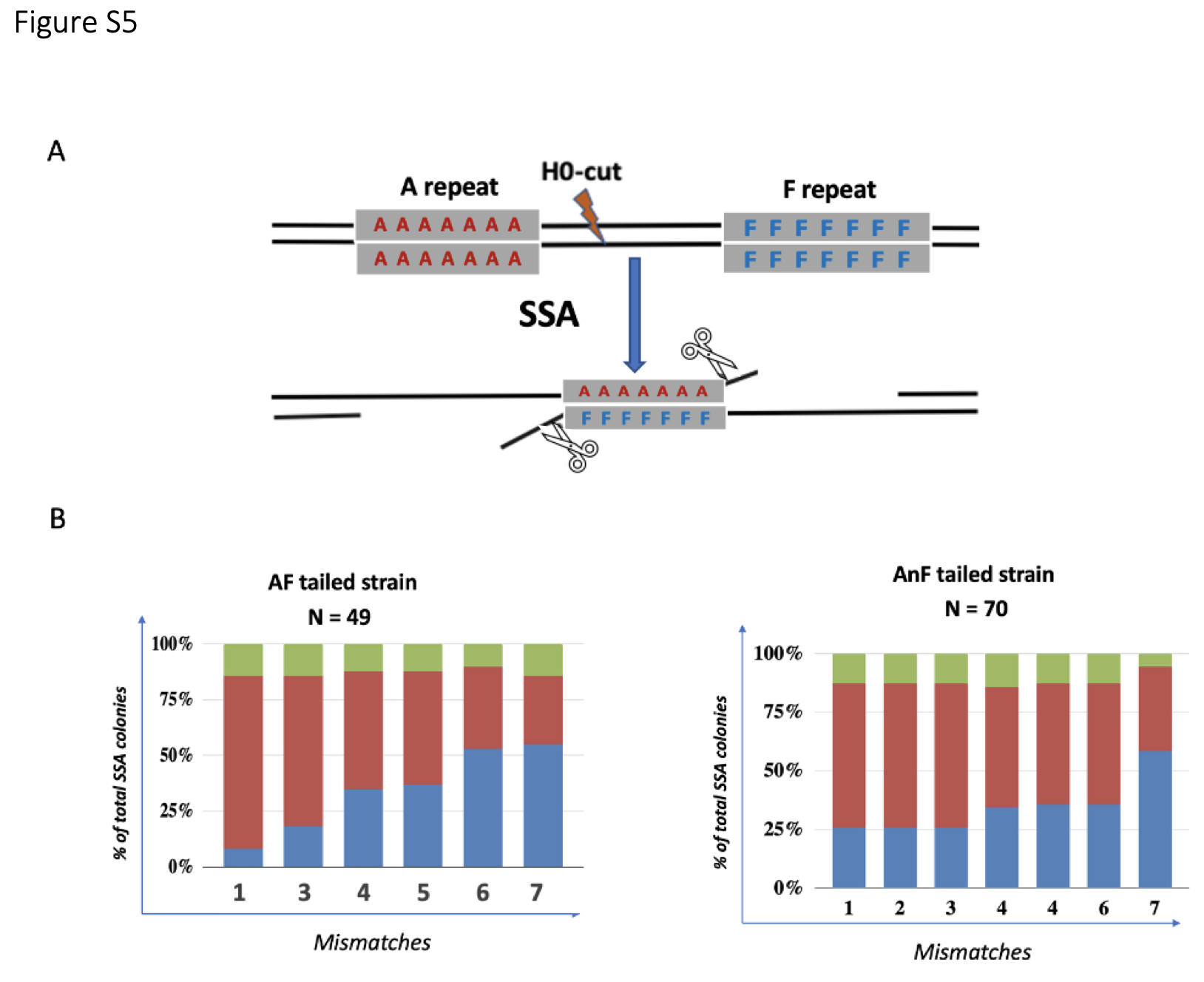

Supplement: S5 Fig — (TIF) [file pgen.1010527.s012.tif]

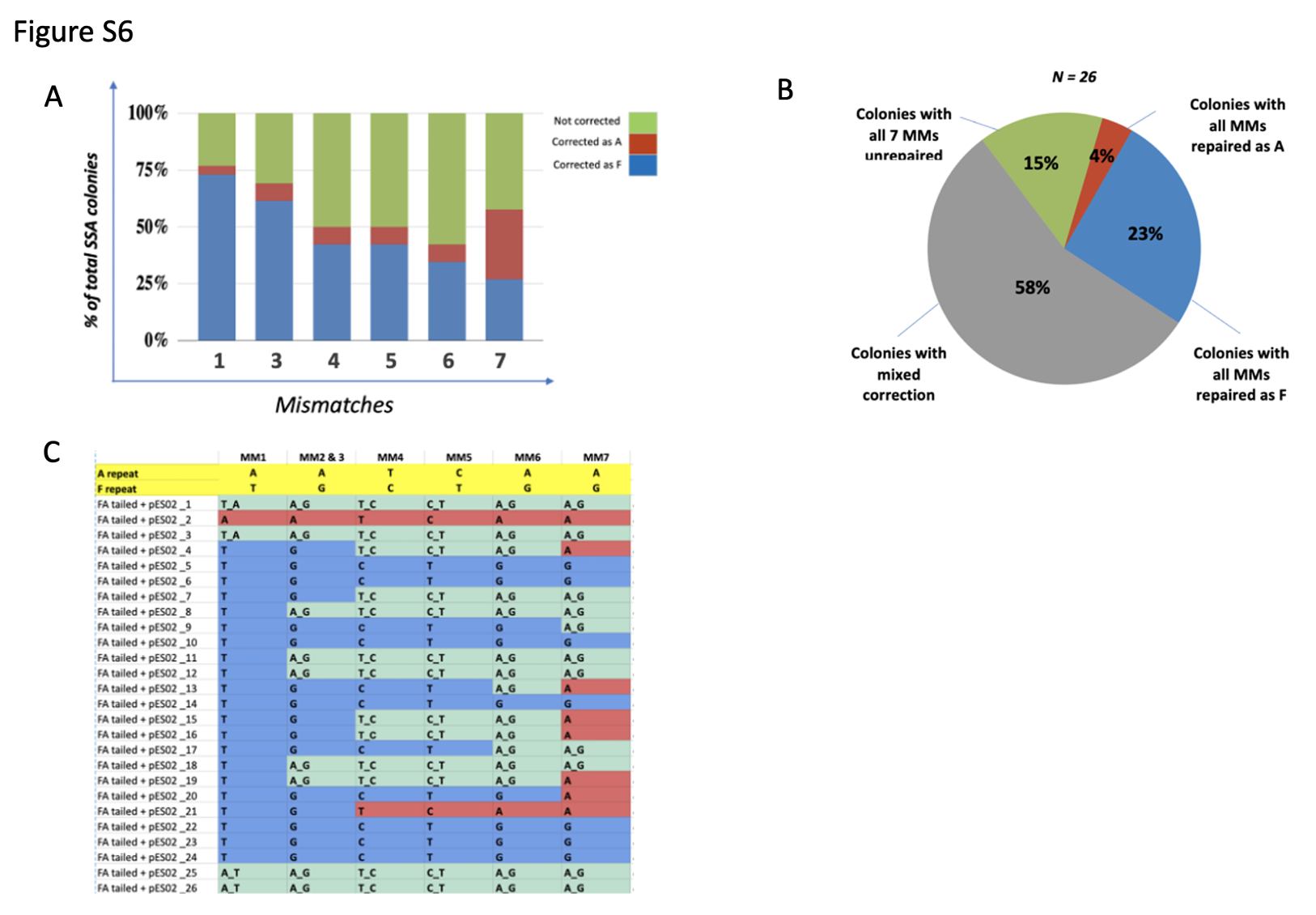

Supplement: S6 Fig — (TIF) [file pgen.1010527.s013.tif]

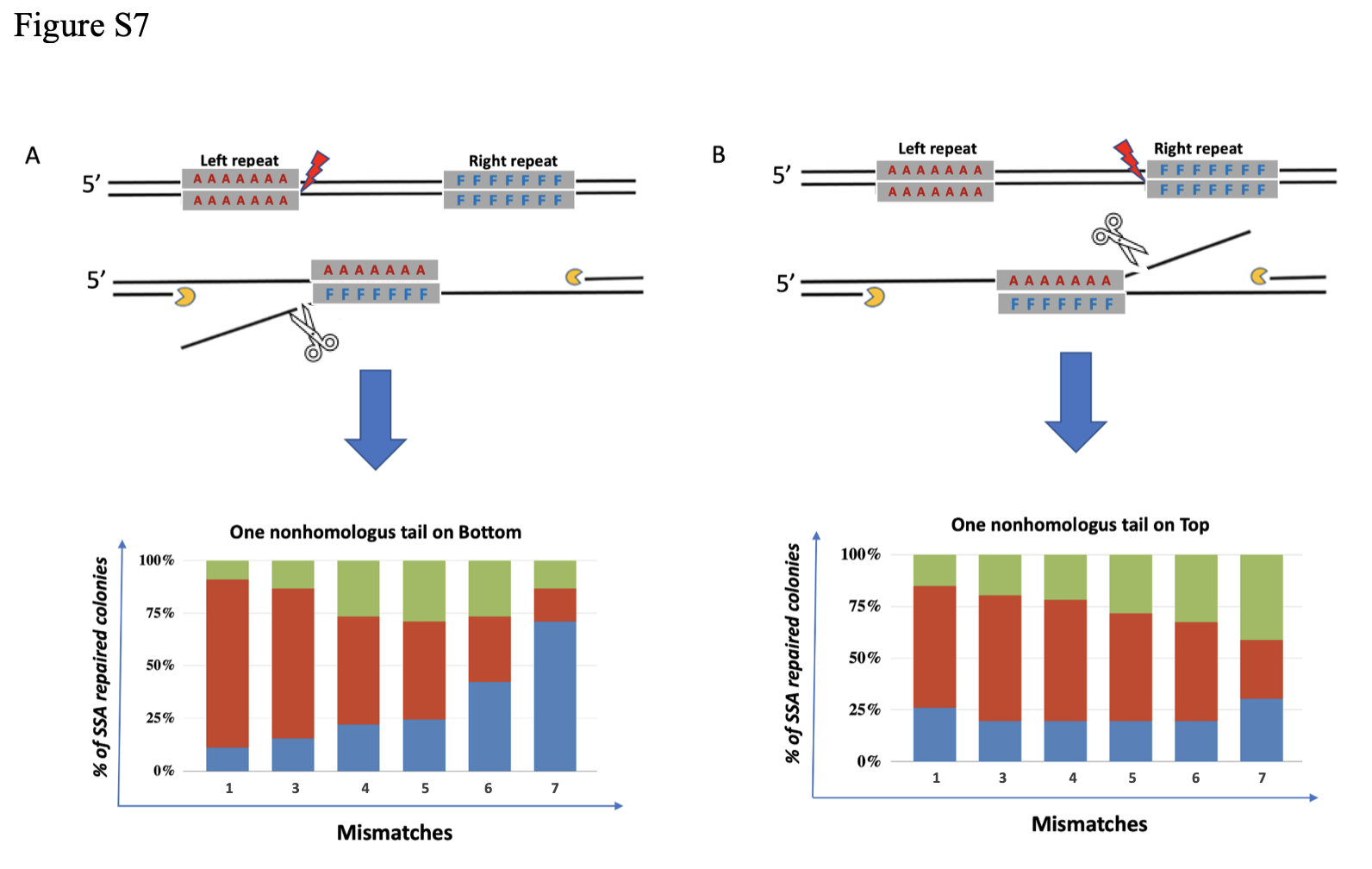

Supplement: S7 Fig — (TIF) [file pgen.1010527.s014.tif]

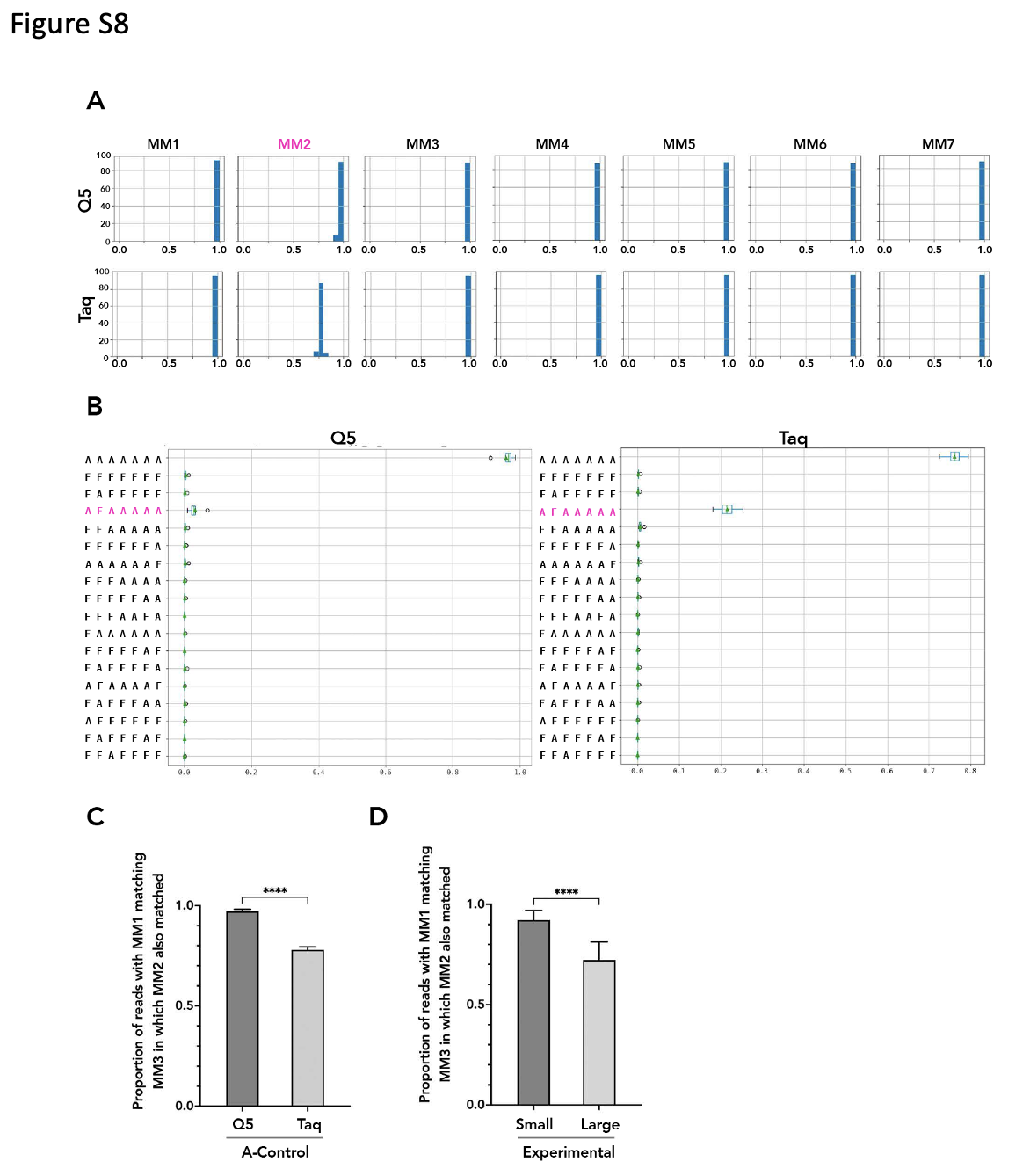

Supplement: S8 Fig — (TIF) [file pgen.1010527.s015.tif]

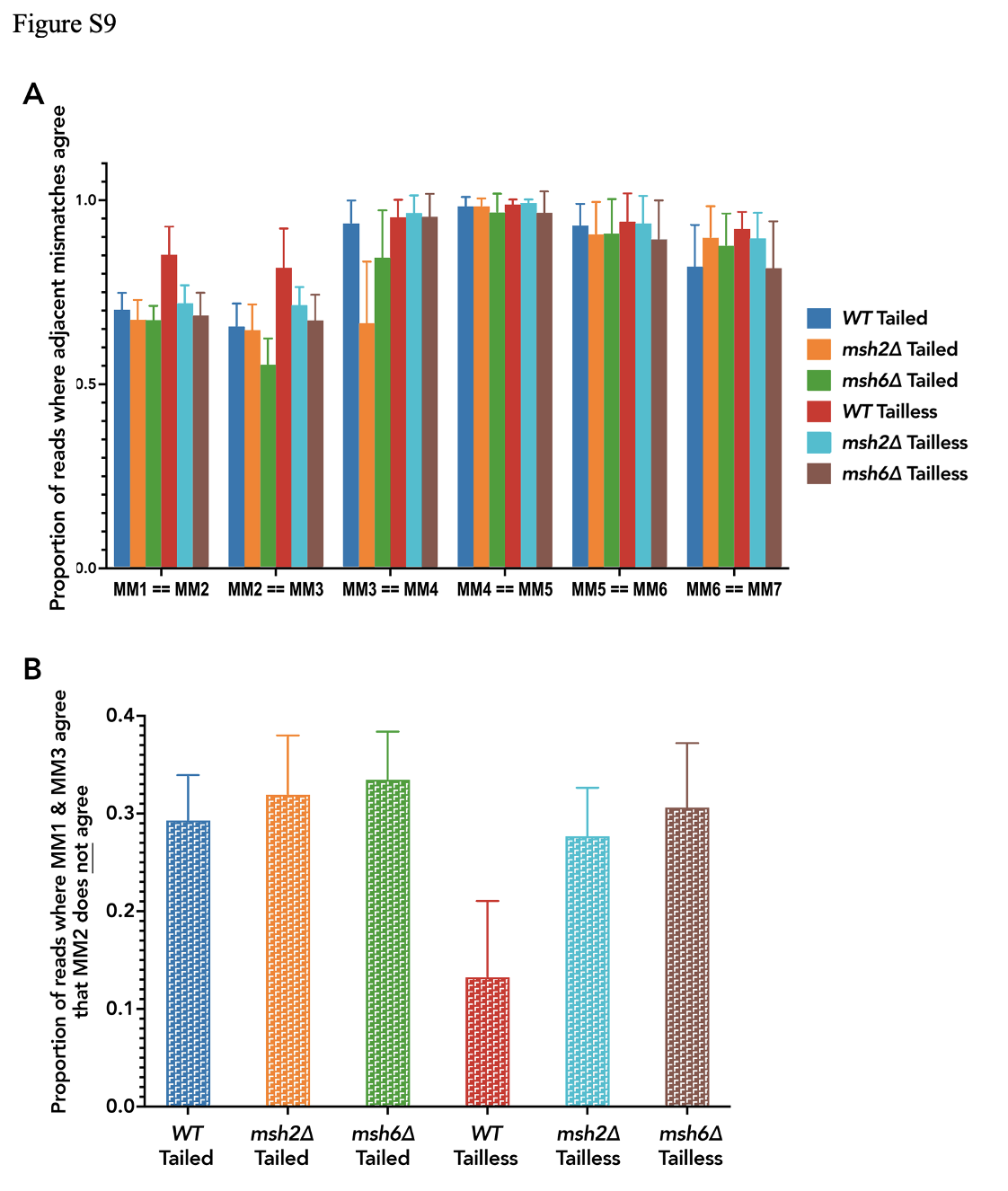

Supplement: S9 Fig — (TIF) [file pgen.1010527.s016.tif]
