# Supplementary material for: Nonhomologous tails direct heteroduplex rejection and mismatch correction during single-strand annealing in Saccharomyces cerevisiae
Source: PLoS Genet. 2024 Feb 5;20(2):e1010527. doi: 10.1371/journal.pgen.1010527 (PMC10868807; doi:10.1371/journal.pgen.1010527)
Supplement: Attachment — Submitted filename: Response to Reviewers 12-12.pdf [file pgen.1010527.s017.pdf]

Response to Reviewers

Reviewer's Responses to Questions

We have endeavored to respond to the Reviewers comments below. We note that we have added a significant amount of new work, first to address questions of the bias of the mismatch repair, but also to document our discovery that the central role of Rad52 annealing activity is largely dispensable in SSA when there are no nonhomologous tails. We show this both in the chromosomal system involving 200-bp repeats and in a plasmid-based assay. This is a fundamentally important finding in its own right and adds substantially to this revised paper.

Reviewer #1:

Comments

1. There is a lot of information presented in this paper; Table 1 alone required a serious effort. In general, the observations are quite interesting though at least for this reviewer there is a sense that the mechanism of mismatch correction in the different constructs has not been fully explored, though it would not be trivial to do so. Also, the authors wrote the paper as if the presence of a non-homologous tail impacting rejection is a new concept. The new work presented is not diminished by the previous studies. With this said, the authors should more clearly state in the introduction the previous work showing that the presence of tails during genetic recombination impacts rejection.

We have more carefully referred to previous work concerning the role of nonhomologous tails in provoking rejection, including papers by Alani's and Jinks-Robertson's labs.

2. Throughout the paper, assays utilizing HO endonuclease and Cas9 endonuclease with a variety of guide sequences are compared to each another. This comparison is predicated on the near perfect efficiency and identical kinetics of cutting for each Cas9/guide RNA combination. Any less than perfectly efficient guides could inflate cell survival in this assay. The manuscript does not provide evidence to confirm that each guide behaves in the same manner. There is evidence to show in other systems that Cas9 cleavage efficiency varies widely between guide sequences (Doench et al. Nature Biotechnology 2016, 34:184, among many others), as well as a review of efficiency prediction algorithms for guide RNAs (Haeussler et al, Genome Biology 2016 17:148). To remedy this, the authors should analyze DSB formation upon galactose addition either by Southern Blot, comparing each unique guide RNA to each other and to the original HO endonuclease. I recognize that Cas9 expression is continuous when cells are grown in galactose, but there are examples where even under continuous selection for Cas9 expression cleavage is not efficient (e.g. Bao et al. ACS Synth Biol, 2015, 4:585). At the very minimum a Southern blot time course for the tailless substrate presented in Figure 2D should be done because the choice of a guide RNA in this situation is quite limited.

We have endeavored to show that Cas9 cleavage is generally efficient at the sites we have used, although in some instances there may be a small delay. Most importantly, we have directly compared the cleavage by Cas9 at two sites very close to the HO cleavage site and found comparable results. One of these two sites (DSB-2 – see Figures 2C and 4A and B) is the same sequence as used in the Tailless construct. The results with Cas9 and with HO are in agreement.

We have endeavored to show that Cas9 cleavage is generally efficient at the sites we have used, although in some instances there may be a small delay. By plating on galactose plates we are employing a continuous induction which should minimize differences in the kinetics of cutting by

Cas9s. Most importantly, we have directly compared the cleavage by Cas9 at two sites very close to the HO cleavage site and found comparable results. One of these two sites (DSB-2 – see Figures 2C and 4A and B) is the same sequence as used in the Tailless construct. The results with Cas9 and with HO are in agreement.

We believe that we are making the appropriate comparisons. We now compare Cas9-cut tailless strains with Cas9-cut tailed strains instead of comparing it with Gal::HO cut tailed strain.

In Fig 3 we compare Gal::HO cut mutants vs Gal::HO cut wt; likewise we compare Cas9 mutants vs Cas9 WT strains.

Finally, we monitored our Cas9 strains by assaying induced-colonies by PCR to ascertain the fraction of cells receiving DSBs. Almost all cells undergo DSB-formation when Cas9 is expressed. In the early stages of this work we identified some Cas9 strains where Cas9 appeared to cut inefficiently. These strains gave a significant fraction of DSB-surviving-colonies that retained the parental configuration instead of the SSA product. These strains were discarded but we note that they were characterized as having increased viability and uncorrected mismatches at all of the sites. Where appropriate, we have acknowledged that certain results, such as uncorrected mismatches, may be due to delayed cutting by Cas9. These results could also be due to a failure to correct heteroduplex DNA.

Additionally, manuscript presents evidence, both in terms of viability and sequencing results, that the Gal::HO endonuclease and the constitutively-expressed Cas9, in pAB101, that targets MATa behave similarly (Fig 2b).

3. Figure 3B and Table 1: I am curious why the effect of a msh3 knockout wasn't tested for the tailless divergent strain. Such an experiment would answer if the decreased viability seen for the msh2 knockout divergent tailless strain resulted from a defect in MSH2-MSH3 function (that could be explained in a model) or some other function that relied only on MSH2 (perhaps damage signaling?).

The *msh3* data are now included along with *msh2 msh6* and *msh3 msh6*. We find that *msh3 msh6* has a mismatch correction pattern comparable to *msh2*, but there are differences in the efficiency of repair that we have not identified.

4. To better understand nature of the positional and biased mismatch repair effects shown in Figures 4A, B, S4, and S5, the authors could invert either the entire F-A construct in the chromosome or just the non-homologous DNA sequence. I recognize that a bias of repair was still seen in the tailless strain (Figure 4C), but this bias appeared weaker than seen for the tailed strain. The inversion experiments could really address what I feel is one of the most interesting aspects of the work.

As the reviewer notes, the persistence of the bias in the tailless strain suggests that the origin of the bias, is probably not within the SSA spacer DNA. Inverting the cassette may not provide much additional information in this case. The reviewer also argues that the deletion of the spacer in the tailless strain results in a weaker F>A bias, relative to the tailed strain. We point out, however, that If

we disregard the “uncorrected” fraction and only focus on the F:A ratio, the bias towards the F fragment in tailless strains is roughly equal to that in the tailed strains.

Although we have not inverted the intervening sequences, but we have created DSBs at various sites, as well as comparing cases where the arrangement of repeats is AF instead of FA. We note that there are preferential repair events occurring at the left end in both cases. We considered whether these events could be attributable to 3’ to 5’ exonuclease (proofreading) activity of DNA pol δ as we have shown in Break-Induced Replication assays, but showed that this activity is not responsible for the bias. These new data are shown in Figure 6. It is not possible to test the role of Rad1-Rad10 and related proteins directly, as the flap cleavage activity is required, whether cleaving at the “normal” end of the annealed region or at a more interior site, as shown in Figure 8C.

Minor comments

1. The authors note that repair in the identical repeat tailed strains was lower compared to the identical repeat tailless strain. The decreased viability seen in the identical repeat tailed strains was seen previously in plating assays (for example, Goldfarb et al. Genetics 169:563) but not in Southern blot analysis (Sugawara PNAS 2004, 101:9315; Goldfarb Genetics 2005, 169:563). It’s probably worth indicating the disconnect between the genetic and physical assays, perhaps when the authors discuss the possibility of daughter cell survival loss.

When using the Gal::Cas9 our current data in Fig 2C shows that the tailed AA strains have a survival frequency in the vicinity of 80 to 100% (Fig 2C). Various published Southern blot measurements put this value at 77%, 92% and 105% (Sugawara MCB 2000, Sugawara PNAS 2004, Goldfarb Genetics 2005) so the Cas9 data lines up with the Southern data.

The viability results using Gal::HO plasmid, however, result in somewhat lower values. We suspected that this has to do with some peculiarity with this plasmid, where the Gal10::HO promoter is placed adjacent to the centromere in the plasmid pFH800. Since the Gal promoter is bidirectional it may weaken the centromere and destabilize the plasmid, a phenomenon previously documented. We have tested the wildtype tailed strains using a different Gal::HO plasmid (Fig. 2) and we observe values closer to the Gal::Cas9 values.

2. Page 4, line 11. It is worth briefly mentioning that MutL homolog proteins have been implicated in heteroduplex rejection in other systems (Rayssiguier, Nature 1989, 342:396; Hum NAR 2019, 47:4554). The Hum and Jinks-Robertson study is particularly interesting; their experiments were performed in wild-type, msh6 (mismatch recognition) and mlh1 (mismatch processing) strains containing homologous and divergent substrates that allowed them to distinguish between crossover and non-crossover events. Their major observation was that mismatch recognition and processing could impact different kinds of recombination events involving divergent DNA sequences.

We discuss Hum et al.’s results and note how ours differ.

3. Methods section, “Analysis of SSA” and “Galactose-inducible Cas9 Endonuclease pRT02”

In the first section “Analysis of SSA” the DSB induction process is described as “HO endonuclease…. was induced by addition of 2% galactose to create a single DSB between the 200-bp repeated segments [10]. After induction of GAL::HO nearly all survivors were SSA products and could thus be monitored by colony counting. Cells were plated for individual colonies on YEPD and on YEP-GAL plates to induce endonuclease.”

In the second section, “Galactose inducible Cas9 endonuclease pRT02”, DSB induction is described “to induce the Cas9 endonuclease activity, cells were grown for 3h in YP-lactate, and then ~500 cells were plated on YEPD and YEP-Gal plates. This second section could be clarified. Does this mean

that the Cas9 induced DSBs and the HO induced DSBs were treated differently in the SSA survival assays? If so, could we see a control to show that this difference has not affected SSA efficiency?

The methods section has been changed to say that the Gal::Cas9 strains were induced in the same way as the Gal::HO strains. Both are plating assays (gal vs dextrose plates) followed by replica plating to the appropriate marker carried on the plasmid. The protocols are in fact the same.

The first paragraph above could be interpreted to mean that we added galactose to a liquid culture which we did not. This paragraph was re-written.

4. Figure 2 Panel A. Please specify that the tailed reaction involved GAL-HO and the Tailless GAL-CAS9. It's vaguely written in the legend.

We have changed the text.

5. Figure 4B. Please indicate above the graph that this was performed in FA tailed strains.

Done

Reviewer #2: Major comments:

Overall, I am enthusiastic about the subject area of this manuscript, and the authors' conclusions, if true, would be very interesting. Frankly there are a lot of observations, some intriguing mechanistic hints, but the current manuscript seems to be lacking a many key experiments that are necessary to understand what is actually going on.

1. The key problem is the fact that the tailless construct is cut using Cas9, whereas the tailed construct is (most commonly) cut using HO. This makes it impossible to determine whether the differences that the authors are ascribing to the nonhomologous tail might actually be due to differences in the kinetics of Cas9 expression, DNA binding, and/or cleavage. Given that the role of the non-homologous tail in heteroduplex reject might entirely be due to timing, it's crucial that the same nuclease be used for both tailed and tailless constructs. Unfortunately, this means the authors need to repeat many of their experiments with Cas9 cleavage. For some experiments (e.g. Fig 4) this has been done. For others it is simple (e.g. simply cut the tailed AA strain with Cas9 in Fig 2A). But for other experiments, this will require substantially more work (e.g. Figs 3,5,6).

We have now shown that using the exact same Cas9 sequence that was used in the Tailless strain, but inserted close to the site of HO cleavage, yielded the same results as HO. Hence there is not a concern that Cas9 outcomes differ from HO cuts. We feel that we are making the proper comparisons. For example, in Fig 3 we are comparing Gal::HO cut mutants vs Gal::HO cut wt; likewise we compare Cas9 mutants vs Cas9 WT strains. In Fig 5 we compare the HO DSB strains (panel A) separately from the Cas9 DSB strains (panel B).

We also made a direct comparison between Gal::HO induced and constitutively-expressed Cas9 inductions. We examined the variations between HO and Cas9 for a majority of the mutants, which were tested in both tailed and tailless strains using inducible and constitutive assays (Supplementary Table 2). The viability rates were comparable, with p-values above 0.05. In general, the findings demonstrated that the gRNAs utilized in the investigation caused DSBs similar to those

created by HO cutting. However, these assays did not reveal much additional information concerning the bias.

2. Another unresolved problem with this manuscript is the fact that the repair biases are not understood. Repair in the Tailed SSA construct is biased towards the F repeat; when the repeats are swapped (Fig S5) repair becomes biased towards the A repeat. The authors conclude that "The bias to correct sequences in favor of F is in fact a bias in favor of whichever allele is located close to the left end of the annealed structure." (pg. 14). But "close to the left end" really doesn't really explain anything. Is this the asymmetry of the cut relative to the repeats? Does resection of the region between F and A expose strand-specific secondary structures? Is the bias due to something much more prosaic—for example the perfect homology at the 3' end of the repeats is far longer than the perfect homology at the 5' end? (The 5' end has clustered sequence variants, see Fig S4 but a better diagram of this in Fig 1 is probably needed.)

To address the bias, we have done several things. First we have made it clear that much or all of this bias is independent of Msh2 (or Msh3 Msh6). Second, as described for Reviewer 1, we asked if asked if Polδ's proofreading activity could be responsible for the Msh2-independent bias at the left end, and ruled out this attractive mechanism. We can't directly test the only alternative we have imagined: that sometimes flap removal in the region where the mismatches are more dense clips off some of the sequences that should have annealed; but this seems likely to us. We note that we also looked at the bias where there is not a 1-bp indel, by converting it to a mismatch.

Additionally, we have shown that the bias remains even after removing the spacer region in the tailless strain, which rules out the possibility of secondary structure in the spacer causing the bias.

3. The authors claim that differences in survival in Fig 2D between the various tailed intermediates argues that the presence of even a single flap stimulates heteroduplex rejection (pg 11). This is one of the major arguments of the paper. However, the authors cannot argue this solely with data from F-A strains. To prove that this survival effect is due to heteroduplex rejection, they must repeat the experiment in A-A strains and show that the effect of the flaps is restricted to strains where heteroduplexes can form.

We have investigated this point further and find that a single flap is intermediate between two tails and no tail, but more like the Tailless construct. We show data both for AA and FA.

4. The authors speculate about the role of the DNA polymerase delta proofreading activity on the correction of mispairs in this study (see for instance Fig 6, Fig S7). This really seems at odds with the effect of MMR defects (see Fig 5A, for example) where this sort of repair ought to be the _only_ mechanism for repair, yet there is no bias for the correction of MM6 or MM7 in msh6 or pms1 strains and unrepaired heteroduplexes predominate. These data suggest that the role of the proofreading activity is much more subtle and should actually be tested using appropriate pol3 point mutations.

This has been done. Pol3-01, which eliminates the chewing back of the invading 3' end in BIR does not affect the bias seen here.

5. The model the authors propose for mismatch correction in the heteroduplex is really intriguing. They argue (pg 19) that removal of the non-homologous flap creates a bias for the repair of that

strand analogously to the use of strand discontinuities in MMR. Unfortunately, it appears that their own data are not consistent with the model. In supplemental figure 7, asymmetric cleavage to generate a one-flapped intermediate does not bias the repair to the flapless strand as this model would predict. A slightly modified model that would argue for blocking of the unflapped 3' end with DNA polymerase might suggest that the flapless strand should be treated as the continuous strand and the Rad1-Rad10-cleaved flapped strand would then be the one repaired. In either case, the data in supplemental figure 7 doesn't show the sort of symmetric change in bias that this model predicts.

We agree that the data does not show a symmetric change to the gradient that would be expected from the model when the DSB is on one side or the other. It is possible that the effects may be obscured by the presence of uncorrected mismatches. We have modified our discussion.

Other comments:

1. Some changes to Figure 1 (and related figures) will probably improve the comprehension of the assays.

We have made extensive changes to Figures and hope we have improved their clarity.

A. The authors should include the MATa site between the F and A repeats in Fig 1A like they do in Fig 2B so that it is clear that nothing has changed about the strain.

Fig 1A was modified by adding the 117bp MATa sequence as suggested. Fig 2C was also modified to show a close up of where Cas9 cuts.

B. The authors need to indicate the distance between the cut site and the F and A repeats (particularly due to the biased repair in the tailed assay). Based on Fig. 2B, it appears that F is closer to the DSB than A.

The DSB is closer to F than to A. Figure S1 shows the distance between the DSB and F when Cas9 is used (268bp). When HO is used the distance is 253bp.

C. The authors are missing an opportunity to make the Figure 1 (and later figures) really clear when they use different colored "X" characters to indicate the sequence variants between the F and A repeat. Why not use "F" instead of "X" for the F repeat and "A" instead of "X" for the A repeat? These make the diagrams match the outcomes in Fig 6 (for example) and make the figures understandable when printed in black & white.

Thank you for the suggestion. Figure 1 was revised accordingly.

D. The authors depict several SSA outcomes. In their "mixed repair" product "a", they still have a heteroduplex that is not pointed out as in the unrepaired product "d". The difference between "a" and "d" is that some sites in "a" are repaired, whereas none are in "d". This should be clarified.

We have endeavored to differentiate between heteroduplex arising from a failure of mismatch repair from a mixture of genetically distinct outcomes if more than one repair event occurred.

E. The authors probably should also drop the word “sectored” from their repair product “d”. Although it is true that the colonies are heterogeneous and are made up of distinct sectors after the original heteroduplex-containing strain divides, the word “sectored” implies use of a colony color sectoring assay which isn’t being used here and could be confusing. Perhaps “genetically heterogeneous colony” is what is really meant here (and is also relevant to “a”).

We agree that they may well have been sectored colonies at the beginning but those distinctions have been lost by taking all the cells in the colony together. We included the term genetically heterogeneous colony (as a product of mixed correction) in the manuscript. Also, we have explained that there probably were sectors, but by the time we analyze the colony the results are – as noted by the reviewer – “mixed.”

F. There is a fifth SSA outcome that they authors do not depict here. In Fig. 6 it’s clear that there are colonies with repair in which no heteroduplex is retained (e.g. the MM1,2,3 repaired as F and MM4,5,6,7 repaired as A). This outcome should be added.

We haven’t tried to show every outcome, but in Fig. 6 (now 7), only the most representative outcomes. As for the outcome mentioned, we clearly showed FFFAAAA (6th genotype from the top).

2. The authors should compare their distribution of SSA outcomes from the NGS experiment in Fig. 6 with the outcomes derived from Sanger sequencing. Do these results give similar percentages at each site for lack of repair/repair to G/repair to A?

We have added more comparison of NGS and Sanger sequencing. In general there is good agreement but there are more unrepaired markers in the NGS set, which may reflect delayed Cas9 cleavage in the particular experiment from which these data are derived.

3. The way the genotypes are displayed in Fig 6A are really inconsistent. In the cases where initial heteroduplexes were observed, they show both strands. In cases without heteroduplexes, they only show one strand. The author should show both strands in all cases for consistency.

Understood. We now show both strands

4. The authors should drop the analysis of MM2 throughout their data set. It’s clear that they don’t trust it (rightfully) due to the high frequency of deletion formation in long homopolymer runs in PCR (see their methods for “correcting” the data). The discussion regarding this variant (and Fig S8,9 and Fig 6 BC, which I believe must be from “uncorrected” sequencing data??) unnecessarily complicate the manuscript, as the status of MM2 does not play an important role for understanding the results.

We acknowledge the problems caused by the presence of Ts in the homopolymer run. In the Sanger sequencing analysis we grouped MMs 2 and 3 together as a simplification so that there are only 6 scored markers. We could, however, score all 7 markers in the NGS sequencing, as we describe.

However, while we recognize the limitations of the homonucleotide data, we strongly believe that it is crucial to include it in the manuscript. The existence of homonucleotide runs poses a significant challenge for analyzing DNA sequences, and our findings can provide valuable insights for future studies focused on investigating diseases that involve nucleotide repetitions. We have

thoroughly addressed the potential issues associated with this result and provided a detailed explanation of the problems encountered during our analysis. As such, we believe that this result is essential to the overall scientific contribution of our research and should not be excluded from the manuscript.

5. The effects of the MutS homolog deletions in this assay are quite baffling and complicated by the different roles of Msh2-Msh3 and Msh2-Msh6 in the assay. Moreover, the fact that the msh2 deletion is very different than the msh3 msh6 double mutant is a really intriguing observation. The authors should really include the analysis of a msh2 msh3 double mutant (and possibly a msh2 msh3 msh6 triple mutant if the msh2 msh3 double mutant doesn't look like the msh2 and msh2 msh6 mutants) for the tailed assay in Fig 5 and Fig S3 and increase the number observations of the msh3 msh6 and msh2 msh6 double mutants. The authors infer that the msh2 spectrum is a failure of both MSH3- and MSH6-dependent tail removal and mispair correction pathways, but given the difference between all of the mutants, it's not clear why the msh2 spectrum should be different than the msh3 msh6 double mutant spectrum.

We don't think that the results for *msh2* are different from *msh3 msh6* in terms of mismatch repair in the sense that there are still gradients and the F:A ratios are similar. There is some difference in efficiency, however, that appears to implicate some other process that is at play in the absence of Msh2.

6. The authors state that "Evidence that GAL::CAS9 cleavage might be less efficient and possibly delayed…" (pg 14, bottom). There are no experiments showing delay of cleavage. The only argument that they appear to be making is that the presence of both alleles suggests "a delay in Cas9 cutting" (Fig S6 legend). This argument simply makes no sense. Whether or not repair of mispairs occur on the heteroduplex must, by definition, occur after the heteroduplex is formed (involving DSB formation and resection). Unless, of course, the author's amplification/sequencing protocols cannot distinguish between unrepaired heteroduplexes involved in SSA and cells in which no rearrangements occur.

The reviewer is correct in thinking that the statement is based on the presence of both alleles in the colonies. The proposed explanation is that after cells have been plated on YPGal plates, the cells divide once before cas9 is fully active and cuts the DNA. In this case the two daughter cells could repair differently giving rise to a sectored colony. Sectored colonies can also be explained as the result of mitotic segregation of unrepaired heteroduplex DNA.

7. Are the colors of the repeats swapped in Fig. S7 or were these experiments performed on the A-F strain?

In this specific experiment the AF strains were used. Colors are consistent and orientation noted in all cases.

8. Figure S4 and the description of the nFA strain (pg. 13) don't appear to match. In the text the authors claim that they "replaced the deletion of the 1T in the 'A' sequence by inserting a G at position 23". From this description, I would expect that the insertion would eliminate the deletion in the 'A' sequence and shift the 'A' sequences over. However Figure S4 still shows that the modified sequence is the F sequence, not the A sequence (e.g. they "inserted a G at position 23 in the 'F' sequence"). Also, the nFA sequence appears to have a deletion of a 1T so that there is no sequence length difference with A.

The text is now: “in an nFA strain YES34 obtained from the FA strain by deleting the +T in the F allele and replacing the T at position 23 by a G, 5 bp upstream MM3 (Figure S4A and B & Supplementary Table 1).”. This now agrees with Figure S4. See Table S1 for actual sequences.

Reviewer #3: This is a review of PGENETICS-D-22-01303, "Nonhomologous tails direct heteroduplex rejection and mismatch correction during single-strand annealing in Saccharomyces cerevisiae." This study examines genetic regulation of heteroduplex rejection during single strand annealing (SSA) / repeat mediated deletions. SSA is a DNA double strand break (DSB) repair outcome that involves repeats that flank the DSB that anneal to each other, causing a deletion between the repeats and loss of one copy of the repeats. SSA associated with human disease is unlikely to involve identical repeats, and as such how repeat divergence affects the mechanism of these events (i.e. heteroduplex rejection) is an important question in genome stability. The authors describe a straightforward genetic assay for SSA in yeast that enables examination of two key phenomenon 1) effect of DNA tail on heteroduplex rejection, 2) the pattern of heteroduplex/mismatch resolution. DNA tail is varied by placing the initiating chromosomal double-strand break at various distances from the homologous repeats. The pattern of heteroduplex rejection is examined with both pooled sequencing, as well as sequencing of individual clones that enables analysis of sectored colonies. Several interesting findings are shown: 1) heteroduplex rejection (and hence the influence of mismatch repair / MSH6, as well as the SGS1 helicase) requires a DNA tail, 2) for the events that show partial mismatch correction, there is a gradient that "favoring the sequence opposite the 3’ end of the annealed strand," which again is dependent on a DNA tail. Altogether this study provides novel insight into heteroduplex rejection, along with mechanisms of mismatch correction during DNA repair. The results are clearly presented, with limitations of the approach clearly described (e.g. the limitations inherent in the +T mismatch #2). The Discussion is clear and will stimulate research in this area.

The main concern is the presentation of Figure 6 is relatively hard to follow. Two recommendations:

1. To the novice reader, I think the figure legend and "description" of the SSA genotypes may be difficult to understand. I recommend creating a supplemental figure with illustrations for each genotype that shows the heteroduplex, the likely repair outcome, and then the daughter cells. For example, showing the heteroduplex repaired to FFFFFF/FFFFFF then leading to identical daughter cells, and hence not a mixed colony, would help the novice reader. A limitation of this approach is that such illustrations are models that of course might be wrong, but if clearly described as models, I think this clarity will help readers substantially understand the data.

We feel that another figure is not needed.

2. There is a lot of data on a small graph on 6A. Perhaps keep Figure 6A as a whole figure and lengthen it to make the bars thicker, and move 6B/6C to supplemental. Alternatively / in addition, split MSH2 and MSH6 into separate graphs.

We lengthened Fig. 6 (now 7) to widen the bars.

One minor concern:

3. The cartoon with the 7 X's is not to scale, since the mismatches are not equidistant. I recommend a simple figure under Fig 1B that shows a larger version of the 7 X's illustration but the position of the X's roughly to scale. Of course, the positions of the mismatches are in the table in Fig 1B, put it would be great to have this in an illustration to get a visual of the structure, and if the authors use the

same color/X's schematic as the cartoon used throughout the study, it can help reinforce the relative position of the mismatches in the model figure.

We think it is clear from Figure 1 where the heterologies lie. The genomic sequences of the repeated fragments highlighting the distribution of the 7 MMs can be observed in Figure 1C and also in Supplementary Table 1. Figure 1C was re-drawn. Colors are, we believe, consistent throughout.
